# Kidney collecting system anatomy applied to endourology - a narrative review

**DOI:** 10.1590/S1677-5538.IBJU.2024.9901

**Published:** 2024-03-18

**Authors:** Ana Raquel M. Morais, Luciano A. Favorito, Francisco J. B. Sampaio

**Affiliations:** 1 Universidade do Estado do Rio de Janeiro Unidade de Pesquisa Urogenital Rio de Janeiro RJ Brasil Unidade de Pesquisa Urogenital - Universidade do Estado do Rio de Janeiro - Uerj, Rio de Janeiro, RJ, Brasil

**Keywords:** Cakut [Supplementary Concept], Anatomy, Review Literature as Topic

## Abstract

**Objective:**

To evaluate the surgical anatomy of the kidney collecting system through a narrative review of the literature, highlighting its importance during diagnosis and its approach during surgical procedures for the treatment of renal stones.

**Material and Methods:**

We carried out a review about the anatomy of the kidney collecting system. We analyzed papers published in the past 40 years in the databases Pubmed, Embase and Scielo, and we included only papers in English and excluded case reports, editorials and opinions of specialists.

**Results:**

Renal collecting system could be divided in four groups: A1 – kidney midzone (KM), drained by minor calyx that are dependent on the superior or the inferior caliceal groups; A2 – KM drained by crossed calyx, one draining into the superior caliceal group and another draining into the inferior caliceal group; B1 – KM drained by a major caliceal group independent of both the superior and inferior groups; and B2 – KM drained by minor calyx entering directly into the renal pelvis.

Some details and anatomic variations of the collecting system are related to clinical and radiological aspects, particularly perpendicular calyces, interpyelocalyx space, position of calyces in relation to renal border, classification of the renal collecting system, infundibular diameter and the angle between the lower infundibulum and renal pelvis.

**Conclusion:**

The knowledge of intra-renal collecting system divisions and variations as the angle between the renal pelvis and lower infundibula, position of the calices in relationship with renal edge and the diameter and position of the calyces are important for the planning of minimally invasive renal surgeries.

## INTRODUCTION

The incidence of nephrolithiasis has increased in developed and underdeveloped countries, comprehending 10 to 15% on the population (1). In the USA, prevalence of renal stones has increased 37% (2). Recurrence is 15% in one year, 50% in five years and 80% in 25 years, after the first incidence of renal colic (1). In Brazil, hospitalization index related to renal lithiasis was 2:3000 patients per month in 1996. In 2006, this number increased to 6:7000 per month (3). It is estimated that 50% of patients with stone disease with symptoms of obstruction of urinary system will need to be submitted to surgical intervention (4).

Current treatments for renal lithiasis include Shock Wave Lithotripsy (SWL), percutaneous nephrolithotripsy (PNL), and flexible retrograde ureterorenoscopy (FUR) (5, 6). All these procedures to be performed depend on a good knowledge of intra-renal anatomy. The anatomical variations of the angles between the renal pelvis and infundibulum (IPA), the number and diameter of the calices as well the complexity of the lower pole drainage system affect the success rates for each chosen treatment (7-9).

The lower pole stones are of special interest in endourological procedures. The percentage of complete elimination of fragments from the upper pole, middle calix, renal pelvis, and lower pole are 78%, 76%, 84%, and 58%, respectively after SWL (10). Besides the gravity-dependent factor, which would make difficult the elimination of stone fragments, the lower pole collecting system anatomy is important for the retention of fragments after endourological surgeries (11). Nevertheless, the negative effects of lower IPA, infundibular length, and width are critical for SWL (12). Besides gravity-dependent position of lower pole calices, these anatomical features might influence fragment clearance after SWL for lower pole lithiasis (11, 12).

Knowledge of the renal collecting system anatomy and radiological analysis of urinary system is necessary for the performance of renal punctures during percutaneous surgeries and the management of the ureteroscope and access to the calyces during FUR and consequently to the success of the treatment of intra-renal calculi (13-17). Despite the method of choice for treating lower pole nephrolithiasis, it is important to know if the method used for studying the lower pole caliceal anatomy is precise. This knowledge is very important for planning the percutaneous access, for flexible ureterorenoscopy of the lower pole calices, and also for indicating and predicting the success of SWL for treating lower pole lithiasis.

Correlation between the type of collecting system and technical difficulties that may be found in a particular anatomic group may indicate the probable result of the surgery in special of lower pole calculi.

The aim of the present work is to evaluate the surgical anatomy of the kidney collecting system through a narrative review of the literature, highlighting its importance during diagnosis and its approach during surgical procedures for the treatment of renal stones.

## MATERIAL AND METHODS

In this study we carried out a review of the anatomy of the renal collecting system. We analyzed papers published in the past 40 years in the database Pubmed, Embase and Scielo, using the key expressions "Anatomy"; "Kidney Anatomy"; "Shock Wave lithotripsy", "Percutaneous nephrolithotripsy", "Retrograde ureterorenoscopy", "MRI"; "CT"; "Endourology"; and "Endourologic Surgery". We found several papers in these database and we included only papers in English and excluded case reports, editorials and opinions of specialists.

**Figure 1:**
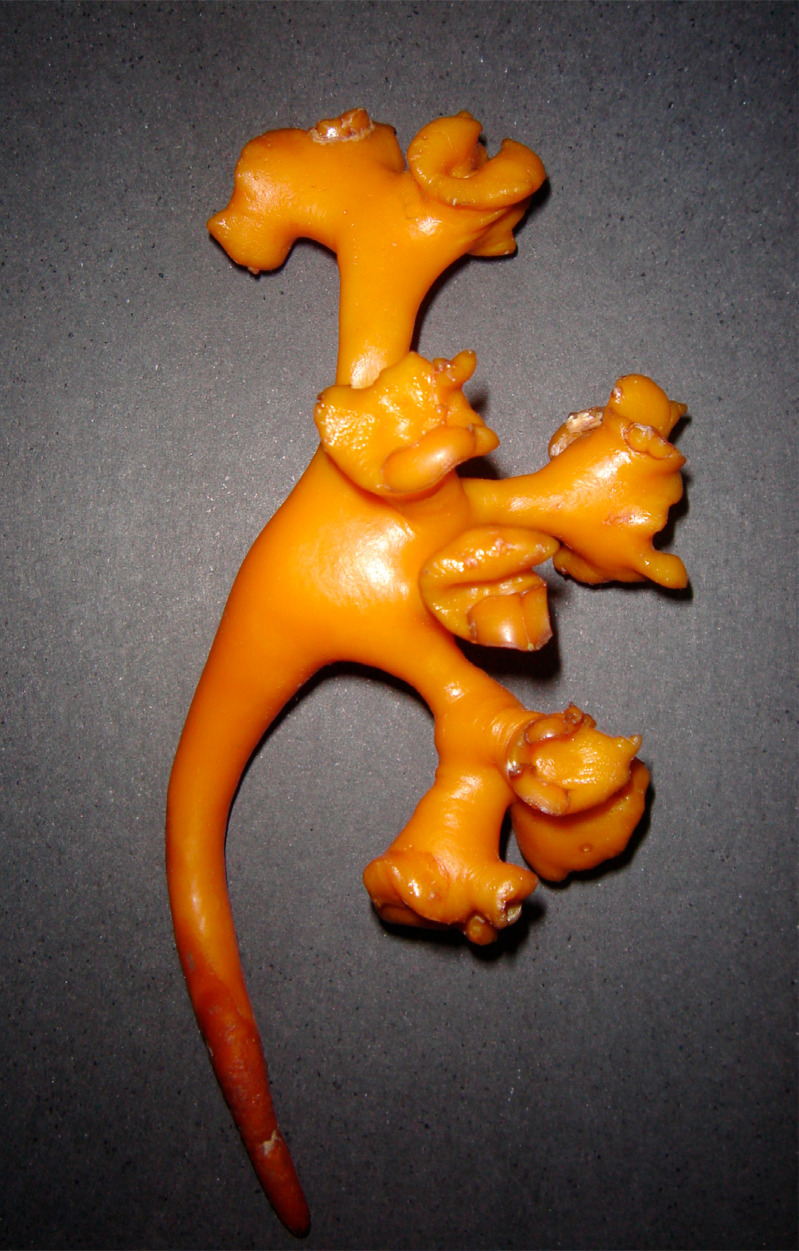
Final aspect of a three-dimensional polyester resin collecting system endocast. We can observe that in this technique the distribution of minor and major calices and the relationships between the renal pelvis and infundibulum is very easy to analyze.

We also studied five renal endocasts of collecting system using the injection corrosion technique with polyester resin (18). In order to perform the injection, we used the following method: for each 100 mL of resin, we added 10 mL of styrene monomer and 2 to 5 mL of catalyzing agent, and the dye (we standardized the following colors: yellow for the collecting system, red for arteries and blue for veins). Following the resin hardening, we initiated the process of corrosion in order to remove all organic material and confection of the mold (Figure-1). After injection, the material was dipped in hydrochloric, sulfuric or muriatic acids for 24 hours. After this time, the mold was removed from the recipient, cleaned and dried for analysis (18, 19).

## RESULTS

### Anatomy of the renal collecting system

Minor calyces drain the renal papillae, and their number are variable: 70% of kidneys present 7 to 9 minor calyces (20). A minor calyx may be simple (when drains one papillae) or compound (when drains two or three papillae). Calyces in the polar regions of the kidney frequently are compound, particularly those located in the superior pole. Minor calyces may directly drain to an infundibulum or get together, forming major calyces that subsequently will drain to the infundibulum; finally, those infundibula, considered primary divisions of the pyelocaliceal system drain to the renal pelvis (19, 20).

Many authors claim that there is only one caliceal infundibulum draining each renal pole (21, 22). However, in a previous study of Sampaio (18, 19), it was shown that in 56.8% of kidneys the lower pole was drained by more than one infundibulum (3 to 7) distributed into two rows: anterior and posterior. In 43.2%, the lower pole was drained by one only infundibulum located in the middle line, that received two or three adhered papillae. The presence of multiple calyces may difficult the drainage, and consequently lower the possibility to eliminate fragments after endourological procedures when compared to a unique calix infundibulum that receives adhered calyces (18, 19).

A recent morphometric study in human fixed corps showed that the accurate knowledge of normal and anatomical variations of pelvicalyceal system is mandatory for urologists as well as radiologists. The intra-renal pelvis was narrow and had funnel shaped appearance in 48.5% of the cases, and the extra-renal pelvis was dilated as balloon shaped in 43 of the specimens. In 20.9% the renal pelvis was partially intra- and extra-renal located. Bilateral symmetry was found in only 27.1% of collecting systems. The length of lower infundibulum was more than 22 mm in 9.7% of cases which directly affects the stone clearance rate during open and endoscopic surgeries on pelvicalyceal system (23).

Knowledge of calyceal pattern is also important for donor selection. Regional anatomy is assessed in detail to decide the precise surgical method, which will avoid donor complication, and to ensure good recipient graft function. A detailed description of calyceal pattern will be of great significance in renal transplantation and also for other urological procedures (24).

A recent systematic review shows the importance of the renal collecting system anatomy applied to endourological procedures. This paper shows that retrograde intrarenal surgery is an effective treatment option for the management of lower pole stones. Infundibular pelvic angle (IPA) seems to be the most important predictor for stone free results. Pelvicalyceal anatomy in conjunction with stone size and hardness seem to dictate the success of retrograde intra-renal surgery for lower pole stones and decisions on the type of surgical interventions (25). The pelvicalyceal anatomical system (PCS) plays a role in both upper calyceal stone formation and in the success of the endoscopic combined intrarenal surgery (ECIRS) procedure (26).

Some details and anatomic variations of the collecting system are clinical and radiologically important, particularly perpendicular calyces, interpyelocaliceal space, position of calyces in relation to the renal edge, the classification of the renal collecting system, the infundibular diameter and the angle between the inferior infundibulum and the renal pelvis (18, 19, 27, 28).

## PRESENCE OF MINOR PERPENDICULAR CALYCES

**Figure 2:**
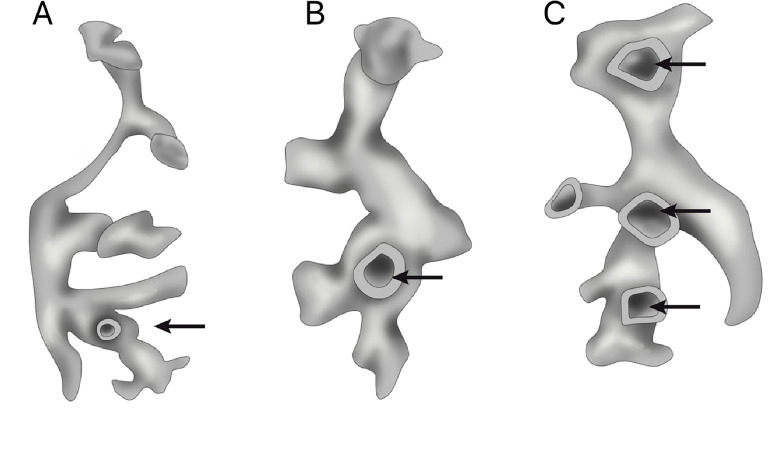
Perpendicular calyces. We can observe in the 3 figures examples of minor calices perpendicular to major infundibulum.

The presence of perpendicular smaller calyces is observed in 11.4% of kidneys when the renal collecting system is studied (18, 19, 27, 28). Perpendicular calyces drain to the renal pelvis or to a major calyx, in general with diameter smaller than 4 mm. Perpendicular calyces are important since they can be overlayed to other structures that difficult radiological visualization and the access during endourological procedures (Figure-2).

## INTERPIELOCALYCEAL REGION

**Figure 3:**
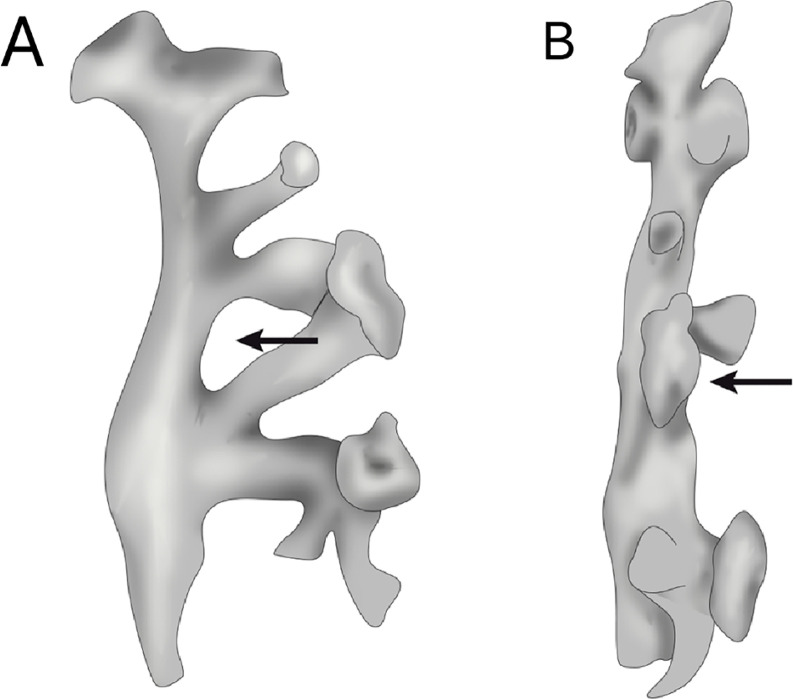
Inter pielo-calyceal region.

Another important anatomical aspect of the renal collecting system is the presence of crossed calyces in the mid-renal zone, present in about 17.2% of kidneys (18, 19, 27, 28). When present, one of the calyces drains to the superior group of calyces and the other drains to the lower calyceal group. Medially, the renal pelvis and the laterally crossed calyces demarcate the region called interpielocalyceal region. This space may present several forms: lozenge (most common), long and narrow, small and round, depending on the size of the calyces (Figure-3). When crossed calyces exist in the mid renal zone, the calyx that drains to the inferior group is anterior in 87.5% of kidneys. This spatial arrangement is important to endourological maneuvers (18, 19, 27, 28).

## CALYCES POSITION IN RELATION TO EDGE OF THE KIDNEY

The position of the calyces in relation to the kidney edge is also important when we review the anatomy of the renal collecting system (27-29). In 27.8% of kidneys the anterior calyces are more lateral (peripheral) than the posterior calyces. In 19.3% the posterior calyces are more lateral than the anterior. In most kidneys (52.9%) the anterior and posterior calyces are overlapped or arranged alternately. Since the local of choice of access of the collecting system is through a posterior calyx during percutaneous nephrolithotripsy, it is important to determine which calyces are anterior and which are posterior. There is a great anatomic variation of the position of calyces in relation to the renal edge. The use of computed tomography with reconstruction is of great importance to the surgical planning.

### Classification of the renal collecting system

The analysis of the renal collecting system proposed by Sampaio is well accepted in the clinical field and is easily identified in the image methods (18, 19).

The intrarenal collecting system is divided in two major groups (with two intermediate variations in each major group). This division is based on the calyceal drainage of the superior pole, inferior pole, and mid-renal zone (hilar) (19). We will describe the anatomy of these two groups, named Groups A and B.

**Figure 4:**
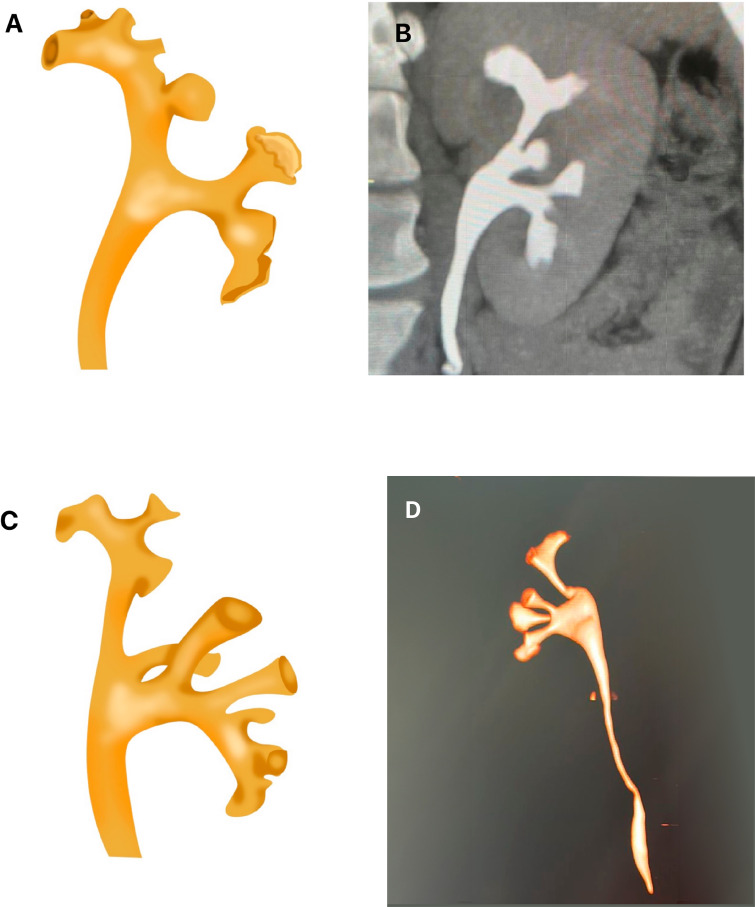
Renal collecting system classification - Group A: In this group, mid renal zone drainage depends on the superior and inferior calyx groups.

**Figure 5:**
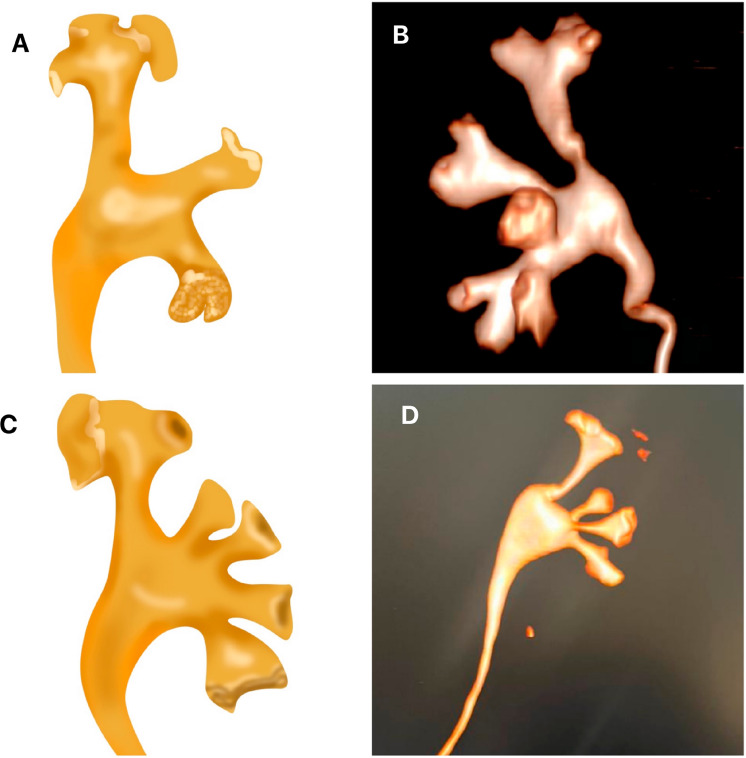
Renal collecting system classification - Group A: In this group, mid renal zone drainage depends on the superior and inferior calyx groups.

**Group A:** It comprises pyelocaliceal systems that present two main calyceal groups (superior and inferior) dividing primarily the renal pelvis, and the drainage of the mid-renal zone depends on these two major calyceal groups (62%) (Figure-4). This group comprehends two different types (variations) of the pyelocaliceal system: types A-I and A-II. A-I type (around 45% of kidneys): the mid-renal zone is drained by minor calyces that are dependent on the superior or inferior calyceal groups, or both, simultaneously (Figure-5). Type A-II (around 17% of kidneys): the mid-renal zone is drained by crossed calyces; one drains to the superior calyceal group and the other to the inferior group, simultaneously (Figure-4).

**Group B:** It comprises by pyelocaliceal systems that drain the mid-renal zone independently of the superior and inferior groups (38%) (Figure-4). This group also includes two different types (variations) of the pyelocaliceal system: type B-I (21.5%) and type B-II (16.4%). Type B-I: the mid-renal region is drained by a major calyceal group, independently of the superior and inferior calyceal groups (Figure-4). Type B-II: the mid-renal zone is drained by minor calyces (one o four) that penetrate directly into the renal pelvis. These minor calyces are independent of the superior and inferior groups.

It is easy to evaluate several important parameters utilized during renal stones surgeries when this classification is used. These parameters include: the frequency of each collecting system type, the number and spatial orientation of calyx, the angle between the lower infundibulum and renal pelvis (LIP), the angle between the lower infundibulum and the inferior minor calyx (LIICA), the inferior infundibular width and length and perpendicular calyx frequency (18, 19). Although this pyelocaliceal classification includes all morphological types of renal calyces and pelvis, it is important for the urologist to keep in mind that the anatomy of the renal collecting system is widely variable.

A recent study proposed a variation of the classification of the renal collecting system including 5 types of renal pelvis: Type A - a single pelvis without bifurcated branch and subclassified into three subtypes according to the cross- sectional area of renal pelvis: Type A1 - If the cross-sectional area ratio of the pelvis to the UPJ is lower than 4 times, the pelvis is considered to be type A1, which is a slimline pelvis morphology, Type A2 - formed by a typical funnel shaped pelvis and the cross-sectional area of the pelvis is about 4–16 times larger than that of the UPJ, and this subtype is the most frequently observed standard morphology; and Type A3 - subtype that included a broad pelvis morphology forming a large box shaped pelvis with the cross-sectional area ratio of the pelvis to the UPJ greater than 16 times. A divided pelvis with bifurcated branches is seen as Type B and subclassified into two subtypes: Type B1 with the wide and flat lower calyx infundibulum, and Type B2 with the narrow and steep lower calyx infundibulum (30). However, in our opinion, this division proposed by these authors includes some alterations that already are considered by Sampaio (18, 19).

An interesting study shows the importance of the Sampaio’s classification in endourological procedures. The anatomical architecture is a prominent factor in the outcomes of flexible ureteroscopy (FURS) (31). Total stone-free rate (SFR) during the first postoperative month evaluation using non-contrast computerized tomography was 63.6%. The evaluation of the SFR in all subgroup of cases (based on Sampaio classification) showed that SFR was significantly lower in subgroup A2 (30.4%), and significantly higher in subgroup B2. The comparative analysis of the operative length showed that the shortest was observed in Type B1 subgroup cases, and the longest (84.7 ± 25.7 min) in the Type A2 subgroup cases. Even though this length was found to be relatively higher in Type A2 subgroup cases than the others, this difference was not statistically significant. Fluoroscopy time was noted to be shortest in B1 subgroup and longest in A2 subgroup with a statistically significant difference. The calyceal structure of the kidney affects the SFR; therefore, a detailed classification of pelvicalyceal system could improve the outcome, decrease the rate of auxiliary procedures, and prevent complications (31).

## INFUNDIBULAR DIAMETER

The infundibular diameter of the inferior calyces is also important during surgical procedures to treat renal stones. Around 60% of kidneys show inferior infundibular diameter equal or greater than 4 mm and in 40% the inferior calyces present at least one infundibulum with a diameter smaller than 4 mm. Smaller infundibula (smaller than 4 mm) may difficult the passage of fragments following SWL and URL (32-25). On the other side, one single infundibulum with a proper diameter (higher than 4 mm) will facilitate the elimination of these fragments (35, 36).

The presence of multiple calyces may difficult the treatment of renal stones located at the lower pole (35, 36). One interesting previous study showed that the lower pole was drained by 4 or more calyces in 49.41%, with a greater prevalence in Group A kidneys, according to the Sampaio’s classification (33, 34). In that same study the only group with no difference of the number of anterior, posterior or lateral calyces is group A2 kidneys, demonstrating that there is a larger number of anterior and posterior calyces in relation to the other studied groups (37).

## ANGLE BETWEEN THE INFERIOR INFUNDIBULUM AND RENAL PELVIS

The angle of the inferior infundibulum and the renal pelvis is paramount on the drainage of the lower pole. Patients with that angle greater than 90º will drain better, and consequently, eliminate residual fragments easier than those with an angle smaller than 90o (32, 33). When the patient lies in the orthostatic position, the kidneys drained by infundibula with angles larger than 90o will present a reasonable drainage (33, 34). When these anatomical details are considered in patients with lower pole stones (inferior calyces), the radiological study before surgery must be obtained previously in order to determine the correct anatomy of the lower pole collecting system (34). The use of computed tomography or magnetic resonance with tridimensional reconstruction may help determine the exact spatial distribution of the calyces (38).

Based on these images, the urologist may discriminate patients with an unfavorable gravitational position of the lower calyces, and other anatomical variations (multiple calyces, infundibula with a diameter lower than 4 mm, and infundibular angle equal or smaller than 90o) that may difficult the elimination of fragments and the surgical access during percutaneous renal surgery or URL (32-35, 37).

For the evaluation of the angle, two lines are traced. The first unites the central axle of the superior ureter with the central axle of the uretero-pyelic junction. In order to draw the second line, we must consider in which calyx the stone is located. If the stone is located in a calyx whose neck accompanies the axle of the inferior major infundibulum, the second line is drawn through the central axle of this infundibulum. However, if the stone is located in a minor calyx whose infundibulum (calyx neck) does not accompany the central axle of the inferior major infundibulum, the second line is drawn through the central axle of the infundibulum of the calyx in which the stone is located (37, 39, 40).

LIP is one of the most important factors for successful FUR (flexible ureteroscope) results, although there is controversy about the limit considered unfavorable, varying from <30° to <90°, depending on the study (33-36). According to Elbahnasy (12) the LIP> 70° is considered a favorable factor to eliminate calculi from the lower pole. Size and volume of calices are also limiting factors for FUR success, regardless of location (41). Long infundibular length (> 3cm) and narrow width (< 5mm) lead to lower FUR success rates (41).

It has been shown that patients with a long infundibulum and with an acute infundibulum-pelvic angle are more susceptible to a second surgical procedure, however without a higher incidence of complications (36). The presence of these anatomic characteristics will difficult the ureteroscope access and the elimination of calculi. It was speculated that these previous limitations in patients with unfavorable angles could have been associated with the use of older ureterorenoscopes (8, 9 ,11). New equipment are more easily to maneuver and present a better vision in relation to the old ones, improving the results of the surgeon and of the surgery itself (42).

Elbahnasy (12) considered the following favorable factors for the elimination of calculi at the renal inferior pole: infundibulum-pelvic angle > 70o, infundibulum length ≤ 3 mm and infundibulum width > 5 mm. On the opposite, angle < 70o, infundibulum length > 3 mm and infundibulum width ≤ 5 mm are unfavorable factors. Sampaio (27, 28) standardized different values as restrictive aspects for the elimination of calculi of the lower pole: angle < 90o, and infundibulum width < 4 mm. When both angle determination methods are compared, it is realized that in the Sampaio’s method the media of angle is 20,21o (17,87o to 22,74o), bigger that when the Elbahnasy method is used, precisely as the parameter values determined by those methods (12, 27, 28, 37, 40).

Previous studies have shown that angles smaller than 45o (8) and smaller than 30o (38) are unfavorable for the success of FUR. Marroig (40) did not observe the presence of angles smaller than 60º at the pyelograms and in 39% of the kidneys the angles measured 61 to 90o, and in 95% of them the collecting systems were of group B. The collecting system of group B showed a smaller IPA (median 92.71o at group B1 and 80.94o at group B2) than the collecting systems of the group A (median of 113,8o in group A1 and 116,8o in group A2). The difference was statistically significant (p=0.0002) (40).

Most unfavorable angles were observed in group B kidneys, regardless the used method for measure. The collecting systems of the group B kidneys show calyces entering directly at the mid renal zone or through an infundibulum (37, 40). Therefore, the inferior calyces are distributed more inferiorly, closing the IPA, resulting in a factor that difficult the elimination of fragments and the ureteral access (36). However, although in the presence of an unfavorable angle, more than 85% of inferior calyces were accessed by the ureteroscope in group B kidneys, similarly of what was observed in the collecting systems of group A1 (87.50%). The lower success rate was observed in group A2 (63.64%), whose IPA usually was greater than 90o (37, 40).

This predominance of angles greater that 90o in group A2 kidneys could explain the presence of minor calyx that extends superiorly, originating from the inferior collecting system, pulling all collecting system cranially, and whose IPA is the greatest among all groups (116.8o). The group with the higher number of kidneys with angles between 61 and 90o was group B (37, 40). These observations make us wonder if the IPA is an important factor for the treatment of renal calculi using SWL and for the elimination of fragments (8, 12, 43); but necessarily is not a difficult factor for the introduction of the ureteroscope, when we consider an IPA > 60o.

The ureteroscope may reach the inferior infundibulum easily, but the angle of the device must be observed in order to access the minor calyces. Group A2 kidneys present the higher percentage of number of major calyces. They show the more closed IPA angles caudally and present the bigger angles directed superiorly, obliging the ureteroscope to follow a sinuous path in order to reach the more superior calyces of the lower pole (37, 40). This observation by itself could justify the lower accessibility of the ureteroscope in group A2 kidneys. In relation to the inferior infundibulum width, A2 group kidneys showed the higher values compared to other groups.

**Figure 6:**
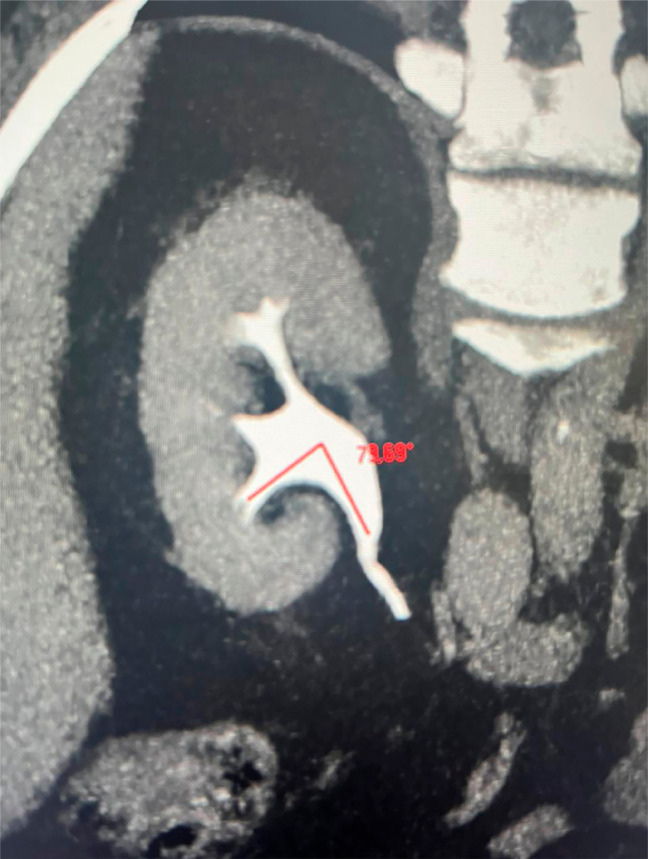
The figure shows a reconstruction of a computer tomography representing the anatomy of the renal collecting system. It shows an example of measurement of the angle between the lower infundibulum and renal pelvis (red line).

Jessen et al. (36) showed that a narrow infundibulum does not affect the success of the ureteroscope treatment, in accordance to the results of the present paper: the ureteroscope access was less efficient in this group, and when the width was a difficult factor for the assessment, it would be expected that the width was the lowest among groups. Therefore, in those studies, infundibular width was not considered a difficult factor related to the success of the ureteroscope treatment (37, 40) (Figure-6).

When we analyze the infundibular length, the measures of group A2 kidneys pointed to lower accessibility of the ureteroscope in this group, however without statistical significance in this sample; the longest infundibular length observed in the molds was present in group A2 (3.09 cm ±0,45 cm) (37,40). Geavlete et al. (8) have already demonstrated that 3 cm of limit of length of infundibulum is a determinant factor for the success of ureteroscopy. Fabregas Arzoz (35) established the limit of 2.2 cm as the length to predict a free stone rate after SWL, similar to the value (2.32 cm) pointed by Jessen et al. (36) that predicted the free stone rate after FUR.

Flexible ureteroscopy has become an essential tool in the arsenal of modern urologists for the treatment of renal lithiasis. Technological advances have made this procedure safe, and efficient, and provided excellent results in the benefit-safety ratio. Some characteristics of the intrarenal anatomy as the infundibulum pelvic angle and infundibular length must be considered before any procedure (44).

### Image exams for planning of renal surgery

In some cases of nephrolithiasis, mainly in the lower pole, the surgical treatment of urinary lithiasis remains a theme of debate (4, 45-47). Tridimensional tomographic reconstruction of the collecting system allows for the previous anatomic knowledge before endoscopic surgeries (45-47). It allows for better decision making of the surgical technique to be employed. With the use of tomographic reconstruction, it is possible to evaluate several anatomic parameters, such as the number of major and minor calyces, their diameters, the angle between calyces and pelvis and between calyces and infundibula, and the position of calyces.

The measurement of angles and length and width of calyces at the preoperative period allows for the use of a lower caliber laser fiber in order to access a calyx with a bigger angulation although these thinner fibers have a lower power of fragmentation. Or the use of a fiber with greater caliber in calyces with anatomic parameters favorable to the introduction of the ureteroscope; these larger fibers support a higher intensity of the laser bean and consequently allows for a faster and more effective fragmentation of calculi (48). The knowledge of the anatomy may also help choose the most proper place for the renal puncture or for the indication of SWL. Knowledge of the anatomy of the renal collecting system and its variants is very important for the surgical planning and interpretation of these exams (40). This is particularly true in lower renal pole calculi. Previous studies proposed that the angle formed between the lower infundibulum and the renal pelvis (i.e., lower infundibulum–pelvic angle [IPA]), the lower infundibulum diameter (ID), and the number of lower pole calyces (i.e., caliceal distribution [CD]) would be the most important factors. The success rate of the treatment of calculi located in the lower pole of the kidney, regardless of the method used, is directly related to the anatomical parameters of this region (37, 40).

A recent study with 145 patients with complex renal calculi treated by FUR showed worse success (83%) when calculi were located in the lower pole (49) due to anatomic factors that difficulted the ureteroscope access, leading to a lower stone free rate (50).

The 3D-HCT is a commonly used examination in the investigation of many renal pathologies such as lithiasis, tumors, vascular anomalies and also in the study of vascular anatomy in renal donors (51-54). The 3D-HCT is much more precise to study calculus location, tumors, and vessels and the lower pole spatial anatomy.

## CONCLUSIONS

The spatial anatomy of renal collecting system is of utmost importance during endourologic procedures. The knowledge of intra-renal collecting system divisions and variations as the angle between the renal pelvis and lower infundibula, position of the calyces in relation to the renal edge and the diameter and position of the calices are of great importance for the planning of minimally invasive renal surgeries.
